# *HuNAC20* and *HuNAC25*, Two Novel NAC Genes from Pitaya, Confer Cold Tolerance in Transgenic *Arabidopsis*

**DOI:** 10.3390/ijms23042189

**Published:** 2022-02-16

**Authors:** Xinglong Hu, Fangfang Xie, Wenwei Liang, Yinhao Liang, Zhike Zhang, Jietang Zhao, Guibing Hu, Yonghua Qin

**Affiliations:** Guangdong Provincial Key Laboratory of Postharvest Science of Fruits and Vegetables, Key Laboratory of Biology and Genetic Improvement of Horticultural Crops (South China), Ministry of Agriculture and Rural Affairs, College of Horticulture, South China Agricultural University, Guangzhou 510642, China; 15238328269@163.com (X.H.); xiefangfang202012@163.com (F.X.); Venwyliang@163.com (W.L.); liangyh131@163.com (Y.L.); poloky2@163.com (Z.Z.); zhaojietang@gmail.com (J.Z.); guibing@scau.edu.cn (G.H.)

**Keywords:** pitaya, genome-wide analysis, NAC gene family, cold stress, genetic transformation

## Abstract

NAC transcription factors are one of the largest families of transcriptional regulators in plants, and members of the gene family play vital roles in regulating plant growth and development processes including biotic/abiotic stress responses. However, little information is available about the NAC family in pitaya. In this study, we conducted a genome-wide analysis and a total of 64 NACs (named *HuNAC1*-*HuNAC64*) were identified in pitaya (*Hylocereus*). These genes were grouped into fifteen subgroups with diversities in gene proportions, exon–intron structures, and conserved motifs. Genome mapping analysis revealed that *HuNAC* genes were unevenly scattered on all eleven chromosomes. Synteny analysis indicated that the segmental duplication events played key roles in the expansion of the pitaya NAC gene family. Expression levels of these *HuNAC* genes were analyzed under cold treatments using qRT-PCR. Four *HuNAC* genes, i.e., *HuNAC7*, *HuNAC20*, *HuNAC25*, and *HuNAC30*, were highly induced by cold stress. HuNAC7, HuNAC20, HuNAC25, and HuNAC30 were localized exclusively in the nucleus. HuNAC20, HuNAC25, and HuNAC30 were transcriptional activators while HuNAC7 was a transcriptional repressor. Overexpression of *HuNAC20* and *HuNAC25* in *Arabidopsis thaliana* significantly enhanced tolerance to cold stress through decreasing ion leakage, malondialdehyde (MDA), and H_2_O_2_ and O_2_^−^ accumulation, accompanied by upregulating the expression of cold-responsive genes (*AtRD29A*, *AtCOR15A*, *AtCOR47*, and *AtKIN1*). This study presents comprehensive information on the understanding of the NAC gene family and provides candidate genes to breed new pitaya cultivars with tolerance to cold conditions through genetic transformation.

## 1. Introduction

Transcription factors (TFs) and *cis*-elements function in the promoter region of different stress-related genes, and thereby alter their expression in response to the stress tolerance [1,2]. Many plant TFs, including MYB, bHLH, AP2, MYC, WRKY, and NAC, have been identified and play diverse functions in various biological processes [3]. Among those TFs, NACs are one of the largest families and play important roles in diverse developmental processes in plants [4,5]. The name of the NAC gene family was derived from the three earliest characterized proteins with a particular domain (NAC domain) from petunia NAM (no apical meristem), Arabidopsis ATAF1/2, and CUC2 (cup-shaped cotyledon) [6,7]. 

Protein sequences of this family reveal that a typical NAC TF has a highly conserved NAC domain with about 160 amino acid residues at the N-terminal region while the C-terminal region is highly diversified in length and sequence, which is considered the transcriptional activation domain [8]. The NAC domain is further divided into five subdomains (A–E) that represent motifs for both DNA-binding and protein–protein interactions [9]. “A” sub-domain functions in the dimerization of the TF, “B”, and “E” have distinctive functions of proteins while “C” and “D” are positively charged and allow the TF to bind to the DNA [10,11,12]. 

The NAC family widely exists in various kinds of plants involving diverse biological processes, including flower formation [13], lateral root formation [14], leaf senescence [15], hormone signaling [16], cell division [17], secondary wall synthesis [18], and fruit growth and ripening [19,20]. NAC TFs have attracted a lot of attention as regulators in plant tolerance during biotic and abiotic stresses such as high salinity, drought, temperature, and pathogen defense [21,22,23,24]. Therefore, the NAC TF family is of great importance for plants to resist harsh environmental conditions. Among these adverse external stimuli, cold stress has become a major environmental factor limiting plant growth and productivity throughout the world [25]. Previous studies have showed that some plant NAC TFs are involved in plant cold stress. Overexpression of *MbNAC25* in Arabidopsis could improve the tolerance to cold and salinity stresses via enhanced scavenging capability of reactive oxygen species (ROS) [26]. Transgenic Arabidopsis plants overexpressing the *GmNAC20* gene enhanced tolerance to salt and freezing stresses. *GmNAC20* may regulate cold stress tolerance through activation of the DREB/CBF–COR pathway [14]. The plasma membrane-associated transcription factor, *ANAC062*, is an important regulator in the cold tolerance signal pathway [27]. Overexpressing *LINAC2* in *Arabidopsis thaliana* could enhance tolerance to cold stress and activated the expression of many cold-responsive genes [28].

Pitaya, a tropical and subtropical plant belonging to *Hylocereus* or *Selenicereus* in the family Cactaceae, is famous for its high nutritional, economic, and medicinal values [29]. Moreover, pitaya can adapt to a wide ecological range such adverse environmental conditions as drought, heat, and poor soil due to it being a kind of succulent plant [30]. Genome-wide surveys of the NAC family have been identified in various plant species, such as Arabidopsis [8], rice [8], maize [31], pepper [32], pear [33], apple [34], and wheat [35]. However, comprehensive analysis of the NAC family in pitaya has not been reported yet. With the completion of the chromosome-level genome sequencing of pitaya [36], it provides a great opportunity to systematically study the NAC family at the genome-wide level. In this study, the NAC family in the pitaya genome were identified and their phylogeny, genomic structures, conserved motifs, chromosomal locations, synteny, and expression levels were analyzed. Transgenic arabidopsis plants harboring *HuNAC20* and *HuNAC25* were studied under cold treatment. The aim of the present study is to identify and validate the candidate *HuNAC* genes involved in cold–stress response that can be used for transgenic breeding to enhance the cold tolerance in pitaya. 

## 2. Results

### 2.1. Genome-Wide Identification of NAC Family Genes

To identify pitaya NAC TF encoding genes, all proteins were annotated from the *H. undatus* genome [36]. In total, 64 NAC genes were identified and named *HuNAC1* to *HuNAC64* according to their chromosomal position. Each of these HuNAC proteins contained an NAM domain (PF02365.15), a specific conserved domain of NAC TF protein family (Supplementary File S1). These genes encoded predicted proteins ranging from 154 to 684 AA (amino acids) with isoelectric point (PI) values ranging from 5.33 to 9.19 and molecular weights from 17.95 to 75.87 KDa (Table S1). Subcellular location of these genes was predicted using an online tool from Molecular Bioinformatics Center (http://cello.life.nctu.edu.tw/) (Accessed on 13 January 2022). The subcellular localization and the protein sequences of the 64 HuNACs were listed in Supplementary File S2. Among the 64 NAC proteins, five (*HuNAC16*, *HuNAC17*, *HuNAC21*, *HuNAC52*, and *HuNAC55*) were predicted to be located in the cytoplasmic; three (*HuNAC3*, *HuNAC34*, and *HuNAC48*) were chloroplast; two (*HuNAC4* and *HuNAC*10) were mitochondrial; and the rest were localized in the nucleus.

### 2.2. Phylogenetic Analyses of NAC Family Members

**Figure 1:**
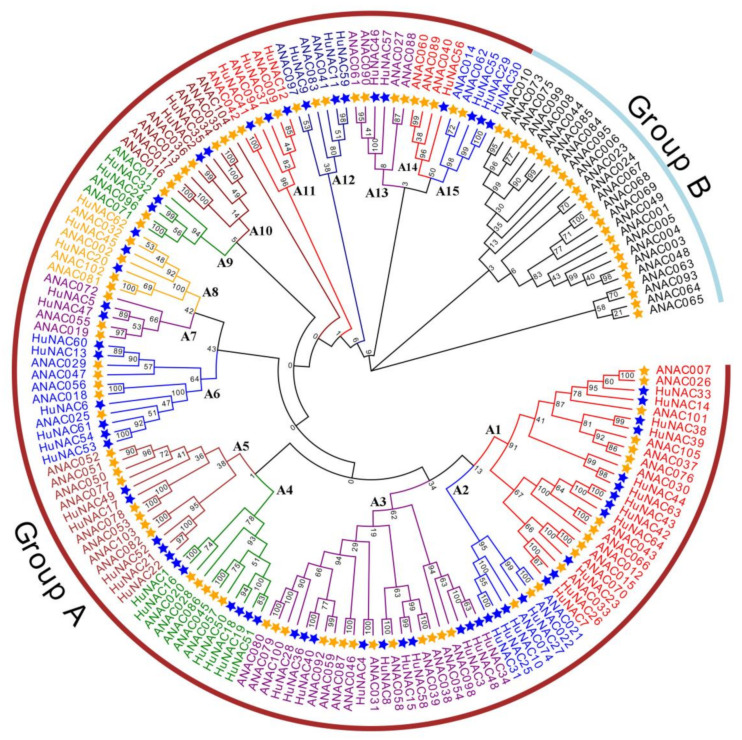
Phylogenetic tree analyses of NAC transcription factor family in pitaya (64 genes) and *A. thaliana* (105 genes). The full-length sequences of the NAC proteins were aligned using ClustalW, and the phylogenetic tree was constructed using the maximum-likelihood method in the MEGAX software. The Bootstrap value was 1000 replicates. HuNACs were indicated by blue stars. All members from both species were designated as Group A (A1-A15) and Group B. Each of the subfamily is indicated in a specific color.

To explore the evolutionary relationships among *HuNAC* genes, a Maximum Likelihood (ML) phylogenetic tree was constructed according to NAC protein sequences from *H. undatus* and *A. thaliana* (Figure 1). Based on the ANAC classification and NAC domain alignments of *HuNACs*, all members of the HuNACs and ANACs were divided into two major groups: Groups A and B. The tree was divided into fifteen subgroups in Group A (A1-15) according to similarities in NAC domain structures. The number of *HuNAC* genes in each subgroup varied greatly. Subgroups A14 and A13 had only one and two genes, respectively. Subgroup A1 constituted the largest clades with 12 pitaya NAC members, followed by Subgroup A3 with 10 sequences. However, no members were detected in Group B in pitaya, suggesting that they might have been lost in these subfamilies.

### 2.3. Gene Structure and Conserved Motif Analyses of HuNACs

**Figure 2:**
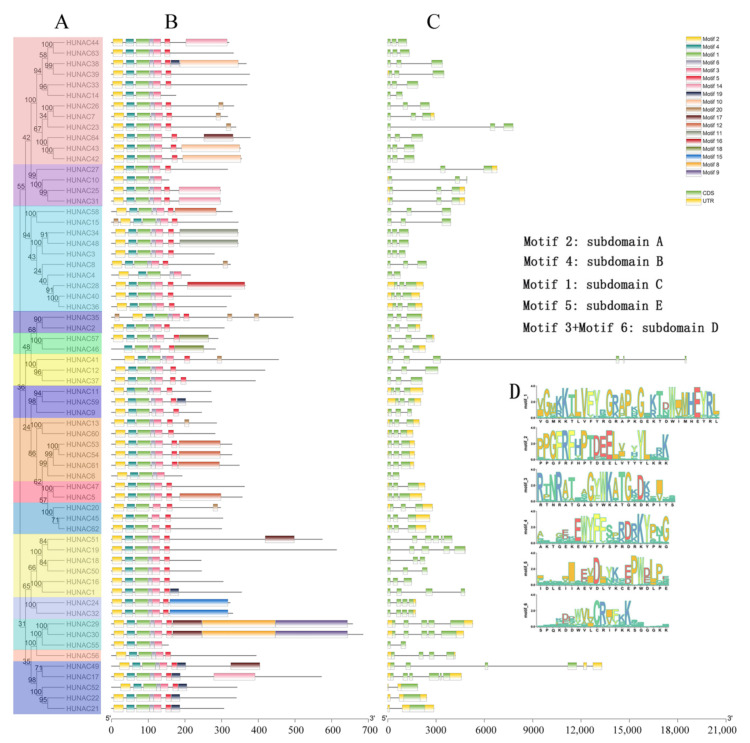
Phylogenetic relationship, gene structure, and conserved motif analyses of 64 HuNAC genes. (**A**) Phylogenetic tree. The phylogenetic tree was constructed using the neighbor-joining method through MEGAX software. The bootstrap analysis was conducted with 1000 replicates. (**B**) protein motif. Schematic diagrams of possible conserved motifs in HuNAC proteins. MEME tool was used to find out the conserved motifs; (**C**) gene structure. The green bars indicate the exons, and the black lines indicate the introns. Yellow bars indicate the UTR region; (**D**) the sequences of key motifs (motif 1, motif 2, motif 3, motif 4, motif 5 and motif 6).

To better understand the similarity and diversity of the *HuNAC* genes, the exon/intron distributions of *HuNAC* and conserved motifs were analyzed. As shown in Figure 2, the 64 NAC genes were divided into 15 subcases. Genes within the same subcase had a similar exon/intron structure in terms of intron number and exon length. For example, among the 12 members in subgroup A3, 11 had two introns, while six genes in subgroup A4 had 2–5 introns.

The conserved motifs of NAC family proteins in pitaya were investigated using MEME online software. Based on this program, 20 distinct motifs were identified (Figure S1). According to frequencies of occurrence, motifs 1, 4, and 3 were the three most frequently presented motifs which were observed in all HuNAC proteins, and most of the conserved motifs were located at the N-terminus. Among the 20 motifs, motif 2, motif 4, motif 3, and motif 5 had the subdomains A, B, D, and E, respectively, and motifs 1 and 6 contained subdomain C (Figure 2D). These results are consistent with the finding that a connection existed between subfamilies and motifs [37].

### 2.4. Chromosomal Localization and Synteny Analyses of HuNACs

**Figure 3:**
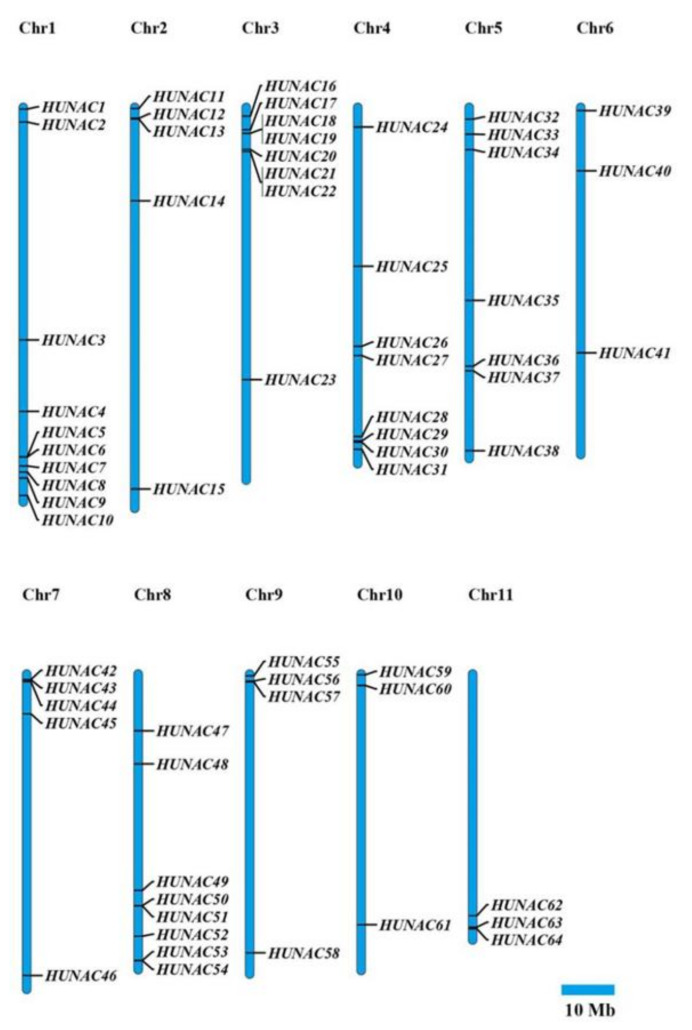
Schematic diagrams of the chromosomal location of the *HuNAC* genes. Eleven chromosomes with varying lengths are shown on the megabases (Mb) scale on the left, and the chromosome number is shown on top of each chromosome.

Genome chromosomal location analyses revealed that the 64 *HuNAC* genes were unevenly scattered on all 11 chromosomes (Figure 3). Chromosomes 1, 3, 4, and 8 contained 12, 8, 8, and 8 *HuNAC* genes, respectively. Chromosomes 6, 10, and 11 had only three *HuNAC* genes. The average numbers of *HuNAC* genes on the chromosome were approximately 6.0.

**Figure 4:**
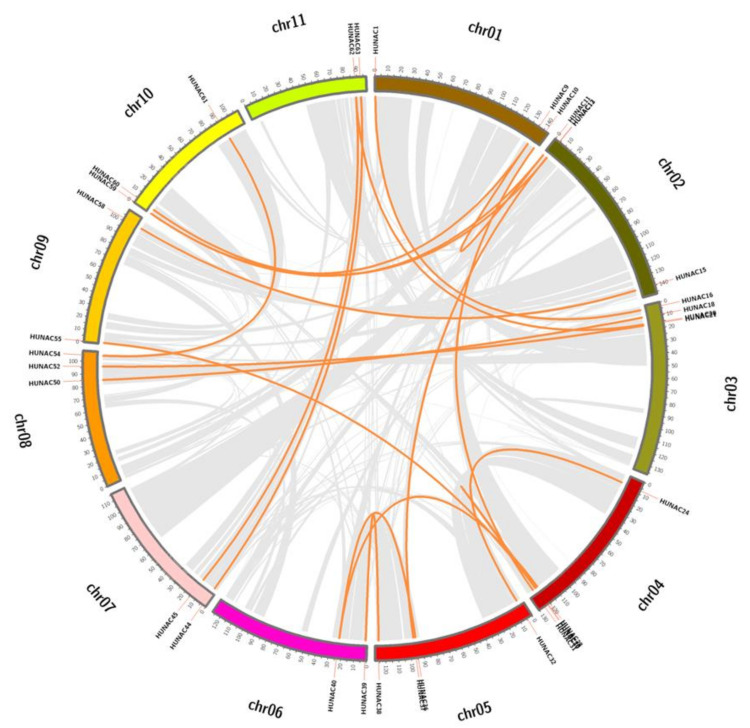
Schematic representations of interchromosomal relationships of the *HuNAC* genes. Gray lines represent all synteny blocks in the pitaya genome, and the orange lines indicate duplicated NAC gene pairs. The chromosome number is indicated at the top of each chromosome.

The phenomenon of gene duplication has been recognized to occur throughout plant evolution, and plays an important role in the expansion of the large gene families in plant [38]. Gene duplication events were investigated to clarify the expansion patterns of the NAC family in pitaya. Twenty segmental duplicated events with 33 *HuNAC* genes were identified in the pitaya genome (Figure 4; Supplementary File S3). The high-identity segmental duplication events in pitaya suggested that this duplication type likely plays a crucial role in the expansion of the pitaya NAC gene family.

### 2.5. Expression Analyses of HuNAC Genes under Cold Treatment

**Figure 5:**
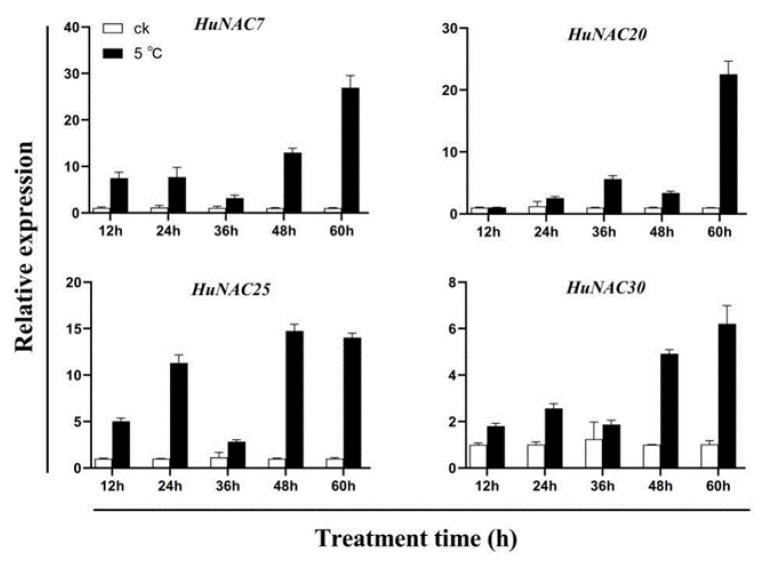
Expression analyses of *HuNAC7*, *HuNAC20*, *HuNAC25*, and *HuNAC30* under cold stress. Three biological replicates were used and bars represent the relative expression of different genes under cold stress.

In plants, many NAC genes are involved in the response to abiotic stresses. Therefore, the expression patterns and putative functions of all 64 *HuNAC* genes were analyzed under cold conditions, and 49 *HuNAC* genes were induced under cold treatment (Figure S2). As shown in Figure 5, compared with normal temperature, higher expression levels of *HuNAC7*, *HuNAC20*, *HuNAC25*, and *HuNAC30* were detected at a low temperature. The expression of *HuNAC7*, *HuNAC20*, *HuNAC25*, and *HuNAC30* showed a trend of gradual increase. *HuNAC7*, *HuNAC25*, and *HuNAC30* were strongly induced at 24 h after cold treatment, while expression levels of *HuNAC20* significantly increased at 36 h after cold treatment. Expressions of *HuNAC7*, *HuNAC20*, *HuNAC25*, and *HuNAC30* reached their maximum levels at 60 h after cold treatment.

### 2.6. Subcellular Localization and Transcriptional Activation Analyses of HuNACs

**Figure 6:**
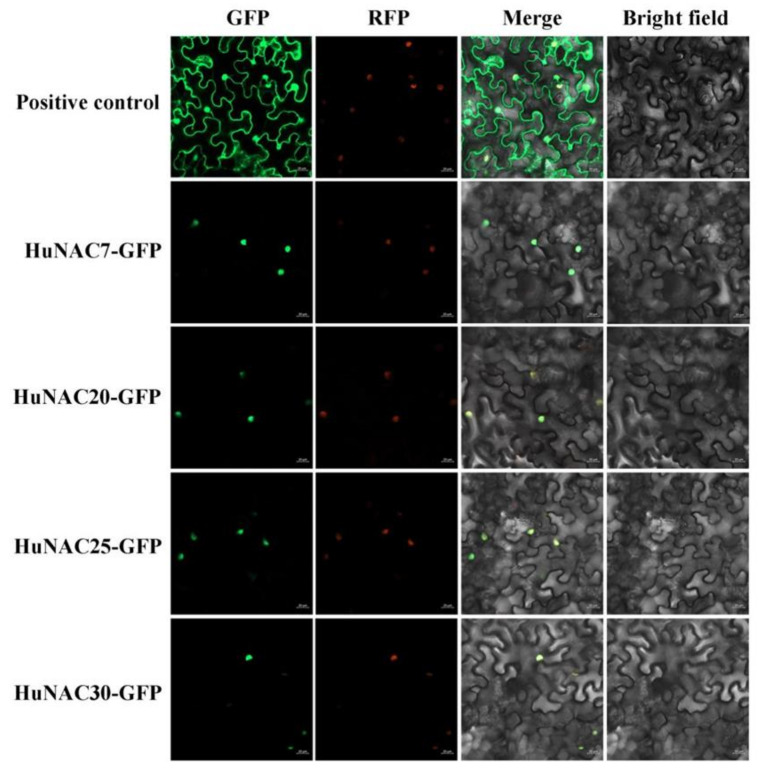
Subcellular localization of HuNACs in *Nicotiana benthamiana* leaves. The fusion protein and GFP-positive control were transiently expressed in *N. benthamiana* leaves by *Agrobacterium tumefaciens* strain GV3101 (pSoup-p19), respectively. Bars = 20 μm.

**Figure 7:**
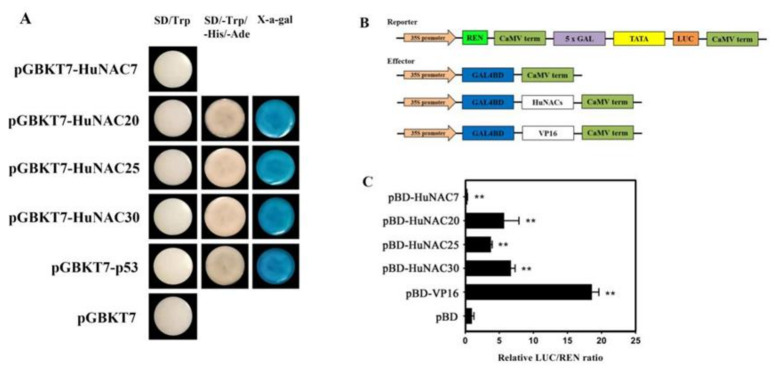
Transcriptional activation analyses of HuNACs. (**A**) transcriptional activation of HuNAC in yeast cells. pGBKT7 and pGBKT7-53 + pGADT7-T were used as negative and positive controls, respectively. Transcription activation was monitored according to growth status of yeast cells and an α-Gal assay; (**B**) diagrams of the reporter and effector vectors; (**C**) transcriptional activation of HuNACs in *N. benthamiana* leaves. The trans-activation ability of HuNACs is indicated by the ratio of LUC to REN. The LUC/REN ratio of the empty pBD vector (negative control) was used as a calibrator (set as 1). pBD-VP16 was used as a positive control. The asterisk indicates a significant difference at the 1% level compared to the pBD.

To analyze the subcellular localization of *HuNAC7*, *HuNAC20*, *HuNAC25*, and *HuNAC30*, their full-length coding sequences were fused with the GFP to construct 35S-HuNACs-GFP vectors, and transiently expressed in leaves of *N. benthamiana*. As shown in Figure 6, the fluorescence of HuNACs-GFP were predominately observed in the nucleus of epidermal cells, while the GFP signal of positive control was detected around the cytoplasm and the nucleus. Those results suggested that the *HuNAC7*, *HuNAC20*, *HuNAC25*, and *HuNAC30*, like the other reported NAC proteins [39], encoded nuclear proteins. Full-length coding regions of *HuNAC7*, *HuNAC20*, *HuNAC25*, and *HuNAC30* were fused to the GAL4BD to generate pGBKT7-HuNACs fusion plasmids to study the transcriptional activation abilities of HuNACs. Like the positive control (pGBKT7-53+pGADT7-T), yeast cells expressing the HuNAC20, HuNAC25, and HuNAC30 grew well on SD/-Trp-His-Ade and showed α-galactosidase activity, indicating that HuNAC20, HuNAC25, and HuNAC30 had trans-activation ability in yeast cells; however, yeast cells expressing the HuNAC7 could not grow on SD/-Trp-His-Ade (pGBKT7) (Figure 7A). Trans-activation of HuNACs were further verified in leaves of *N. benthamiana* using the dual-luciferase reporter system. Compared with the negative control, the expression of the positive control (BD-62SK-VP16), HuNAC20, HuNAC25, and HuNAC30 resulted in a higher value of the LUC/REN ratio. However, HuNAC7 significantly repressed the expression of the LUC reporter in comparison to the negative control (pBD) and the ratio of LUC/REN of HuNAC7 was 0.3-fold compared with the pBD (Figure 7C). These results demonstrated that HuNAC20, HuNAC25, and HuNAC30 are transcriptional activators while HuNAC7 is a transcriptional repressor.

### 2.7. Phylogenetic and Sequence Analyses of HuNAC20 and HuNAC25

*HuNAC20* and *HuNAC25* were cloned based on the sequences of the pitaya genome [36]. The full-length coding DNA sequences (CDSs) of *HuNAC20* and *HuNAC25* were both 891 bp, encoding 296 amino acids with molecular weights of 33.74 and 33.65 kDa and pIs of 7.69 and 6.26, respectively. Homologous analyses of HuNAC20, HuNAC25, and 18 NAC protein sequences from different plant species were aligned with DNAMAN software. The similarity between HuNAC20 and HuNAC25 was 17.7% (Figure S3a). Both HuNAC20 and HuNAC25 had a conserved subdomains (A–E) in the N-terminal region and a diverse activation domain in C-terminal, respectively. HuNAC20 and HuNAC25 both contained a conserved nuclear localization signal (NLS) (Figure S3b,c). HuNAC20 shared 78.2% similarity with CqNAC2 (XP_021772603.1) from *Chenopodium quinoa* and 77.6% similarity with BvATAF2 (QGZ00533.1) from *Beta vulgaris*. HuNAC25 had 67.2% similarity with BvNAC (XP_010679326.1) from *Beta vulgaris* and 64.0% similarity with MeNAC (XP_021630142.1) from *Manihot esculenta*.

### 2.8. Overexpression of HuNAC20 and HuNAC25 in Arabidopsis Enhanced Tolerance to Freezing Stress

To further explore the function of *HuNAC20* and *HuNAC25*, we generated transgenic *A. thaliana* plants constitutively expressing *HuNAC20* and *HuNAC25* driven by the 35S promoter. Homozygous T_3_ lines were obtained on the basis of 3:1 segregation for kanamycin resistance phenotype. Expression levels of the target genes in the homozygous transgenic lines were analyzed by RT-qPCR. Two independent T_3_ transgenic lines with a relatively high expression of *HuNAC20* (NAC20-L1 and NAC20-L3) and *HuNAC25* (NAC25-L2 and NAC25-L3) were selected for cold tolerance experiments.

**Figure 8:**
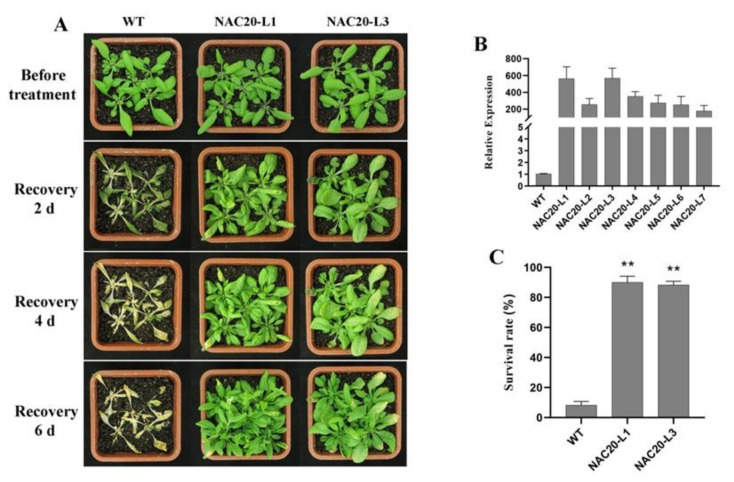
Overexpression of *HuNAC20* in *Arabidopsis* enhanced freezing tolerance. (**A**) Performance and (**C**) survival rates of WT and *HuNAC20* transgenic plants after freezing; (**B**) expression levels of *HuNAC20* in WT and transgenic plants. *AtACTIN2* was used as an internal control. Twenty seedlings per transgenic line were used in each freezing treatment. Data represent average values from three biological replicates (±S.D.). Asterisks indicate significant differences (** *p* < 0.01) between the transgenic lines and WT plants.

**Figure 9:**
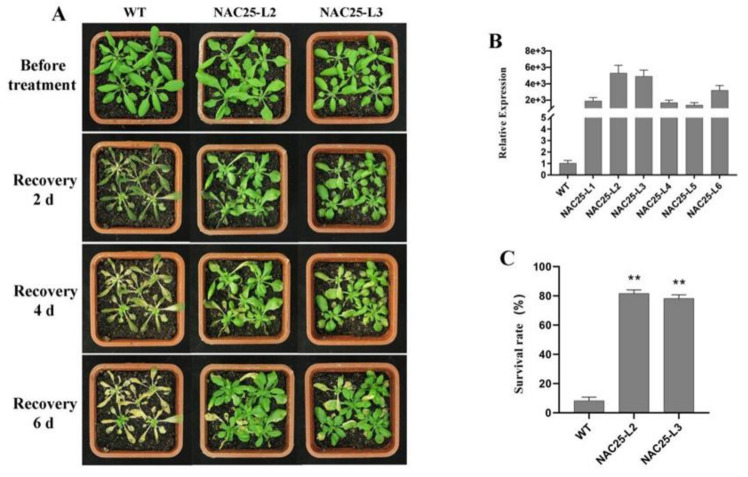
Overexpression of *HuNAC25* in *Arabidopsis* enhanced freezing tolerance. (**A**) performance and (**C**) survival rates of WT and *HuNAC25* transgenic plants after freezing; (**B**) expression levels of *HuNAC25* in WT and transgenic plants. *AtACTIN2* was used as an internal control. Twenty seedlings per transgenic line were used in each freezing treatment. Data represent average values from three biological replicates (±S.D.). Asterisks indicate significant differences (** *p* < 0.01) between the transgenic lines and WT plants.

At normal conditions, no significant difference in morphology was observed between transgenic lines and WT plants. After cold acclimation (48 h at 4 °C) and recovery for 6 d at 22 °C, transgenic plants displayed better performance than wild-type under freezing treatment (−6 °C for 6 h) (Figure 8A and Figure 9A). Most WT plants died with a survival rate at around 8.0%. The survival rates of the transgenic lines (90.0% for NAC20-L1, 88.0% for NAC20-L3, 82.0% for NAC25-L2 and 78.0% for NAC25-L3) were significantly higher than that of WT plants (8.0%) (Figure 8C and Figure 9C). These results indicated that overexpression of *HuNAC20* and *HuNAC25* in *A. thaliana* enhances tolerance to cold stress.

### 2.9. Overexpression of HuNAC20 and HuNAC25 Affected Ion Leakage, MDA Contents, H_2_O_2__,_ and O_2_^−^ Accumulation under Cold Stress

**Figure 10:**
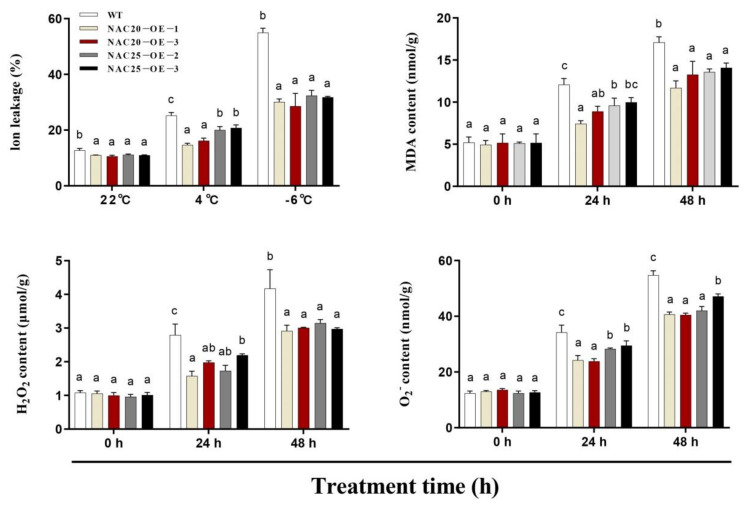
Changes of ion leakage, MDA content, H_2_O_2_, and O_2_^−^ of WT and transgenic lines under cold stress. Ion leakage were determined in WT and transgenic lines under cold stress (4 °C for 48 h) and freezing (−6 °C for 6 h) treatments. MDA, H_2_O_2_, and O_2_^−^ accumulation were determined in WT and transgenic lines under cold stress (4 °C for 48 h)**.** Data represent average values from three biological replicates (±S.D.). The different letters above bars indicate significant differences at the *p* < 0.05 level according to Duncan’s multiple comparison tests.

Ion leakage, MDA content, H_2_O_2_, and O_2_^−^ accumulation are commonly used to assess stress resistance capacity during abiotic stresses. Under normal conditions (22 °C), no significant differences in ion leakage, MDA, H_2_O_2_, and O_2_^−^ contents were detected between the transgenic lines and WT plants. Ion leakage, MDA, H_2_O_2_, and O_2_^−^ contents gradually increased in both transgenic lines and WT plants after cold treatment. The levels of ion leakage in the transgenic lines were significantly lower than those in WT plants during cold (4 °C for 48 h) and freezing treatments (−6 °C for 6 h). After cold treatment for 24 h and 48 h, MDA, H_2_O_2_, and O_2_^−^ contents in the transgenic lines were significantly lower than those in WT plants (Figure 10). These results suggested that overexpression of *HuNAC20* and *HuNAC25* enhances *A. thaliana* tolerance to cold stress by altering the ion leakage, MDA, H_2_O_2_, and O_2_^−^ accumulation.

### 2.10. Overexpression of HuNAC20 and HuNAC25 Activated the Expression of Cold-Responsive Genes under Cold Stress

**Figure 11:**
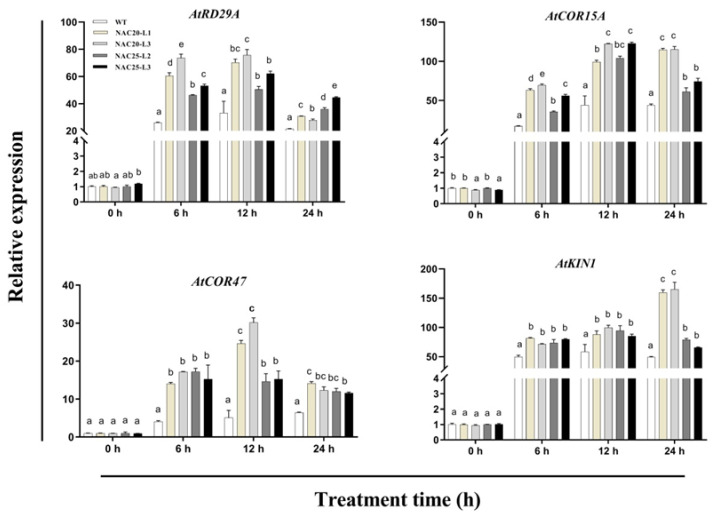
Expression analyses of *AtRD29A*, *AtCOR15A*, *AtCOR47*, and *AtKIN1* in WT and transgenic lines under cold stress. Data represented average values from three biological replicates (±S.D.). The different letters above bars indicated significant differences at the *p* < 0.05 level according to Duncan’s multiple comparison tests.

The transcript levels of cold-responsive genes including *AtRD29A*, *AtCOR15A*, *AtCOR47*, and *AtKIN1* were analyzed in *HuNAC20* and *HuNAC25* transgenic lines and WT plants under cold treatment. The expressions of these genes in transgenic lines and WT plants were relatively low under normal conditions (22 °C). Compared with WT plants, higher transcripts of *AtRD29A*, *AtCOR15A*, *AtCOR47*, and *AtKIN1* were observed in *HuNAC20* and *HuNAC25* transgenic plants after 24 h under cold treatment (Figure 11). These results indicated that *HuNAC20* and *HuNAC25* could activate expression levels of *AtRD29A*, *AtCOR15A*, *AtCOR47*, and *AtKIN1* responsible for stronger tolerance to cold stress in transgenic *A. thaliana* lines.

## 3. Discussion 

NAC is one of the largest families of TFs unique to plants and plays important roles in defense against harsh environmental conditions [40,41]. NAC TFs also play crucial roles in plant physiological processes [42]. With the development of whole genome sequencing technology, great progress has been made in identification of NAC genes from Arabidopsis [8], rice [8], cucumber [24], pepper [32], and apple [34]. However, little information is available about the NAC family in pitaya. In the present study, a total of 64 NAC genes distributed in 11 chromosomes were identified from pitaya (Figure 3). Great differences in the size, sequence, physical, and chemical properties of the proteins encoded by *HuNACs* were detected, which was consistent with NACs from the other plant species [22,24,33,38]. The phylogenetic analyses showed that these *HuNACs* were classified into 15 subgroups with *ANACs* in the Group A, and the subgroup A1 had the most *HuNACs* (19%), followed by the A3 (16%), whereas the A14 subgroup had the fewest genes (2%) (Figure 1). However, Group B had no member of *HuNACs* similar to the results from quinoa [43]. Most *HuNACs* had three exons and two introns (Figure 2), indicating that the genetic structural diversity of NAC in pitaya, which was similar to that of the other species such as kiwifruit [44], *Musa Acuminata* [45], cucumber [24], and white pear [22]. High segmental duplication events resulted in the expansion of the NAC gene family in *H. undatus* (Figure 4), which was consistent with the evolutionary pattern in pineapple [38]. Therefore, we speculated that these pitaya-specific *NAC* genes may have special roles in pitaya.

The growth and development of pitaya are frequently affected by abiotic stresses such as drought, high salinity, or extreme temperatures [46,47]. Cold stress is one of the most important influencing factors restricting pitaya production in South China. Previous studies have suggested that NAC TFs can be induced by cold stress. Overexpression of *SlNAC35* enhanced chilling tolerance of transgenic tomato [48]. Compared with non-cold control fruits, the expression level of *MaNAC1* in the fruits directly stored at 7 °C was significantly increased [49]. *VvNAC17*, a novel NAC TF, was expressed in various tissues under cold treatment and enhanced freezing tolerance in transgenic *Arabidopsis* [50]. In this study, expressions of *HuNAC7*, *HuNAC20*, *HuNAC25*, and *HuNAC30* were strongly induced under cold-stress (Figure 5), indicating that they may be involved in plant responses to cold stress. Subcellular localization is important to elucidate protein function. In our study, HuNAC7, HuNAC20, HuNAC25, and HuNAC30 were nuclear proteins (Figure 6), which is consistent with the other reported NAC proteins [16,39,45].

**Figure 12:**
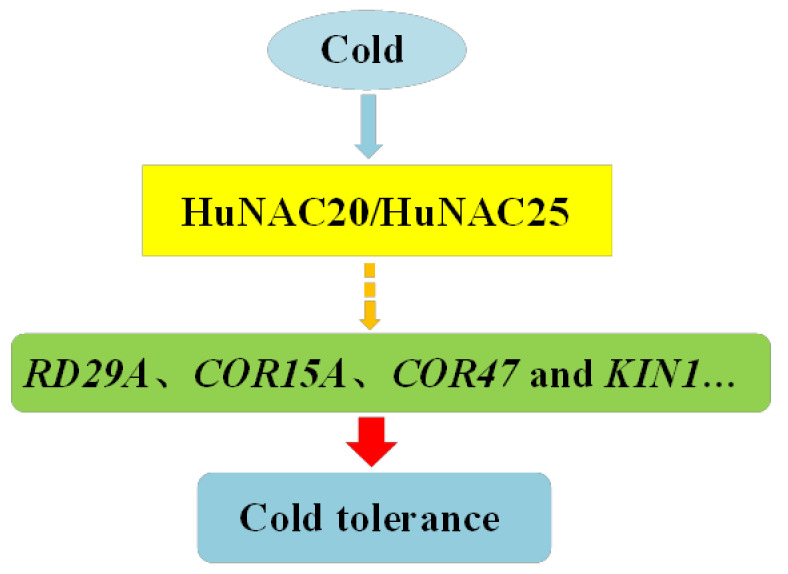
The proposed model of HuNAC20 and HuNAC25-mediated cold stress-responsive signaling. Cold stress induces the expression of *HuNAC20* and *HuNAC25. HuNAC20* and *HuNAC25* regulate the expressions of cold-responsive genes by binding to the *cis*-elements of cold-responsive proteins in their promoters, and then modulate plant tolerance to cold stress.

NAC proteins are involved in plant responses to cold stress. Overexpressing *LlNAC2* in *A. thaliana* enhanced tolerance to cold stress. Compared with WT plants, the transgenic plants had lower electrolyte leakage and higher levels of soluble sugars [28]. Overexpression of *GmNAC20* confers tolerance of freezing stress in transgenic plants by regulating downstream stress-responsive genes such as *cor6.6*, *cor78*, and *cor15A* [14]. Overexpressing *MbNAC25* in *Arabidopsis* increased cold tolerance via enhanced scavenging capability of reactive oxygen species (ROS) under low-temperature stress (4 °C) [51]. In the present study, two novel NAC TFs, i.e., *HuNAC20* and *HuNAC25*, were cloned from pitaya. *HuNAC20* and *HuNAC25* were nuclear-localized NAC-type DNA-binding proteins. Overexpression of *HuNAC20* and *HuNAC25* in *Arabidopsis* enhanced cold tolerance. Ion leakage, MDA contents, and H_2_O_2_ and O_2_^−^ accumulation are commonly used to assess the severity of membrane lipid damage under stress conditions [52]. In our study, overexpression of *HuNAC20* and *HuNAC25* in *Arabidopsis* lines resulted in lower contents of ion leakage, MDA, and H_2_O_2_ and O_2_^−^ under cold stress. Compared with WT, higher expression levels of the cold-responsive genes (*AtRD29A*, *AtCOR15A*, *AtCOR47*, and *AtKIN1*) were detected in the transgenic plants under cold stress (Figure 11). These results indicated that overexpression of *HuNAC20* and *HuNAC25* in *A. thaliana* could activate expressions of *AtRD29A*, *AtCOR15A*, *AtCOR47*, and *AtKIN1* under cold stress (Figure 12). The results are consistent with those NAC TFs from the other plants species such as *Lilium lancifolium* [28], *Solanum lycopersicum* [48,53], and *Malus baccata* [51]. Putative binding *cis*-elements of NAC proteins have been identified to localize in the promoter sequences of these genes [54]. However, the mechanism of interaction between *HuNAC20* and *HuNAC25* and the cold-responsive genes is still not clear yet, and further study is necessary to elucidate it.

## 4. Conclusions

In summary, this study provides the first report on identification and characterization of the *NAC* gene family based on the genome-wide analyses of the *H. undatus* genome. A total of 64 *HuNAC* were identified and divided into fifteen subfamilies. The 64 *HuNAC* were unevenly scattered on all 11 chromosomes. *HuNAC7*, *HuNAC20*, *HuNAC25*, and *HuNAC30* may be involved in cold stress tolerance according to their expression patterns. *HuNAC7*, *HuNAC20*, *HuNAC25*, and *HuNAC30* were nucleus proteins. *HuNAC20*, *HuNAC25*, and *HuNAC30* had transcriptional activity while *HuNAC7* acted as a transcriptional repressor. Overexpressing *HuNAC20* and *HuNAC25* in *Arabidopsis* enhanced tolerance of cold stress by altering the expression of cold-responsive genes in transgenic plants. The results of the present study provide valuable information for a better understanding of NAC TFs involved in cold–stress response in pitaya.

## 5. Materials and Methods

### 5.1. Identification of the Pitaya NAC Gene 

The Hidden Markov Model (HMM) profiles of the NAM domain PF02365.15 were downloaded from the Pfam database (https://pfam.xfam.org/) (Accessed on 13 January 2022). HMM was used to search NAM (PF02365.15) domains from the pitaya genome with values (e-value) cut-off at 1.0 [36]. The integrity of the NAM domain was determined using the online program SMART with an e-value < 0.1. The length, molecular weight, and isoelectric point of each NAC protein were predicted by the online ExPasy program (https://web.expasy.org/protparam/) (accessed on 13 January 2022).

### 5.2. Phylogenetic Analyses of the Pitaya NAC Gene

To investigate the phylogenetic relationship of the NAC gene families in Arabidopsis and *H. undatus*, the Arabidopsis NAC protein sequences were downloaded from the Arabidopsis Information Resource (https://www.arabidopsis.org/) (accessed on 13 January 2022). All NAC TFs were aligned using the MUSCLE set at default parameters [55]. The Maximum Likelihood (ML) phylogenetic tree was constructed using the MEGA7 program [56]. Bootstrapping was performed with 1000 replications. The tree was exhibited by the EVOLVIEW online tool (https://www.evolgenius.info/evolview/) (accessed on 13 January 2022). 

### 5.3. Analyses of Gene Structure and Conserved Motifs in the HuNAC Family

The gene structure of each *HuNAC* gene was drawn using the TBtools software [57]. The MEME (http://meme-suite.org/tools/meme) (accessed on 13 January 2022) was used to identify the unknown conserved motifs with the following parameters: site distribution: zero or one occurrence (of a contributing motif site) per sequence, maximum number of motifs: 25, and optimum motif width ≥6 and ≤200.

### 5.4. Chromosomal Locations and Synteny Analyses of HuNAC Genes 

The chromosome location of each *HuNAC* gene was obtained from the pitaya genome [36]. These data were then integrated and plotted using Mapchart software [58]. The Multiple Collinearity Scan toolkit (MCscanX) was applied to analyze the duplication pattern for each HuNAC followed the operation manual [59].

### 5.5. Plant Material

Stems (6–8 cm in height) in vitro from pitaya cultivar ‘Hongguan No. 1’ (*Hylocereus monacanthus*) on rooting medium (MS containing 0.5 mM IBA) for 15 d were used as materials [60]. The pitaya plants were cultured in a climate chamber at the South China Agricultural University (Guangzhou, China) under a temperature range of 23–25 °C and a 16 h light (50 μmol m^−2^ s^−1^) and 8 h dark. Plantlets were placed in a climate cabinet at 5 °C to analyze the expression patterns of the NAC family. The stems from treatments and control were collected respectively at 12, 24, 36, 48, and 60 h after treatment, immediately frozen in liquid nitrogen and stored at −80 °C until future analysis. 

*Arabidopsis thaliana* Columbia-0 (Col-0) was used for genetic transformation of *HuNAC20* and *HuNAC25*. Transgenic lines and WT plants were grown in 9 cm × 9 cm plastic pots containing a 1:1 mixture of sterile peat soil and vermiculite with normal management in a growth chamber [24 ± 1 °C, 16 h light (50 μmol m^−2^ s^−1^) and 8 h dark and 65% relative humidity]. Seeds of *Nicotiana benthamiana* were planted and cultured under the same conditions. 

### 5.6. Analyses of Ion Leakage, MDA Content, H_2_O_2_ and O_2_^−^

Three-week-old seedlings of transgenic *Arabidopsis* lines and WT were treated at −6 °C for 6 h after cold acclimation at 4 °C for 48 h, followed by 4 °C in the dark for 12 h. The plants were transferred to normal conditions (22 °C) for recovery for 6 d, and then the survival rates were counted. Photos were taken before freezing and after recovery. The rosette leaves were collected for analyses of ion leakage [61], malondialdehyde (MDA) content, and H_2_O_2_ and O_2_^−^ accumulation [62,63]. For chilling treatment, the seedlings were put in a 4 °C incubator, and leaves were collected respectively at 0, 6, 12, and 24 h for expression analyses of cold-responsive genes (*AtRD29A*, *AtCOR15A*, *AtCOR47*, and *AtKIN1*) [64], and *AtACTIN2* (AT1G13320) were used as internal control [61]. Specific primers are listed in Supplementary File S4.

### 5.7. Gene Cloning and Expression Analyses

Total RNA was isolated using the EASYspin Plus Complex Plant RNA Kit (RN53) (Aidlab Biotechnology, Beijing) according to the manufacturer’s protocol. Single-stranded cDNA was synthesized using the PrimeScript™ RT Reagent Kit with gDNA Eraser (TaKaRa, Shiga, Japan). The RT-qPCR primers were designed by BatchPrimer3 (https://probes.pw.usda.gov/cgi-bin/batchprimer3/batchprimer3.cgi) (accessed on 13 January 2022), and the *Actin(1)* reference gene was used as the internal control [65]. RT-qPCR was performed in an CFX384-Real-Time system (C1000 Touch Thermal Cycler, Bio-Rad, CA, USA) using the RealUniversal Color PreMix (SYBR Green) (TIANGEN, Beijing, China). Specific primers are in Supplementary File S4. Each experiment was repeated in triplicate using independent RNA samples. Relative gene expression levels were calculated using the 2^−∆∆C^T method [66].

The full-length coding sequences of HuNACs were cloned using I-5TM2×High-Fidelity Master Mix (MCLAB, San Francisco, CA, USA) with specific primers (Supplementary File S5).

### 5.8. Subcellular Localization Analyses

The full lengths of HuNAC7/20/25/30 without a stop codon were subcloned into the pGreen-35S-GFP vector to fuse with the gene sequence of green fluorescent protein (GFP) (primers are listed in Supplementary File S5). Then, the pGreen- HuNAC7/20/25/30-35S-GFP and the control pGreen-35S-GFP vector were transferred into the *Agrobacterium tumefaciens* strain GV3101 (pSoup-p19), and injected into the abaxial side of 4- to 6-week-old *N. benthamiana* leaves. After 48 h of infiltration, infected leaf tissues were collected for analyses. The GFP signal was captured under a fluorescence microscope (ZEISS LCM-800, Oberkochen, Germany). All assays were repeated three times.

### 5.9. Transcriptional Activation Analyses in Yeast Cells 

The pGBKT7-HuNACs, pGBKT7-p53 and pGBKT7 empty plasmids were transferred into the Y2HGold yeast strain independently using the lithium acetate method (PT1172-1, Clontech) (primers are listed in Supplementary File S5). The transformed yeast cells were cultured on SD/-Trp and SD/-Trp-His-Ade medium. The growth status of yeast cells and the activity of X-α-galactosidase (X-α-Gal) were observed after incubation with 20 mg/mL X-α-Gal for 10–30 min. 

### 5.10. Dual-Luciferase Reporter Assays in N. benthamiana Leaves

For transcriptional activity analyses of HuNACs in *N. benthamiana* leaves, coding sequences of *HuNAC7/20/25/30* were cloned into the 35S promoter-driven pBD vectors to fuse with the yeast GAL4 DNA-binding domain (GAL4BD) as an effector (pBD-HuNACs). The double-reporter vector contained a firefly luciferase (LUC) driven by five copies of the GAL4-binding elements (5×GAL4) and minimal TATA region of CaMV 35S. Renilla luciferase (REN) in the same vector driven by the CaMV 35S promoter was used for normalization. The primers are listed in Supplementary File S5.

### 5.11. Arabidopsis thaliana Transformation and Phenotypic Analyses

The open read frame (ORF) of *HuNAC20* and *HuNAC25* were subcloned into the pPZP6K90 vector under the control of the 35S promoter and introduced into *Agrobacterium tumefaciens* strain GV3101. The recombinant vectors were transformed into Arabidopsis Col-0 using the floral dip method. Positive plants were screened on MS medium containing 100 mg L^−1^ kanamycin and identified by PCR detection. The expression levels of *HuNAC20* and *HuNAC25* in the homozygous T_3_ transgenic lines were analyzed by RT-qPCR, and the primers used were listed in Supplementary File S5. Two homozygous T_3_ transgenic lines were used for cold tolerance experiments and photographed with a digital camera (G16, Canon, City, Japan).

## Data Availability

Data are contained within the article and Supplementary Materials.

## Supplementary Materials

The following supporting information can be downloaded at: https://www.mdpi.com/article/10.3390/ijms23042189/s1.

## Abbreviations

TF	Transcription factor
RT-qPCR	Reverse transcription quantitative real-time polymerase chain reaction
AA	Amino acid
PI	Isoelectric point
kDa	Kilodaltons
MDA	Malondialdehyde
ROS	Reactive oxygen species
NAM	No apical meristem
GFP	Green fluorescent protein
LUC	Luciferase
REN	Renilla
ORF	Open read frame
CDS	Coding sequence
WT	Wild type
COR	Cold regulated
IBA	Indole-3-butyric acid
NCBI	National Center for Biotechnology Information
