# Supplementary material for: HuNAC20 and HuNAC25, Two Novel NAC Genes from Pitaya, Confer Cold Tolerance in Transgenic Arabidopsis
Source: Int J Mol Sci. 2022 Feb 16;23(4):2189. doi: 10.3390/ijms23042189 (PMC8876859; doi:10.3390/ijms23042189)
Supplement: Supplementary file 1 [file ijms-23-02189-s001.zip › Supplement figures.pdf]

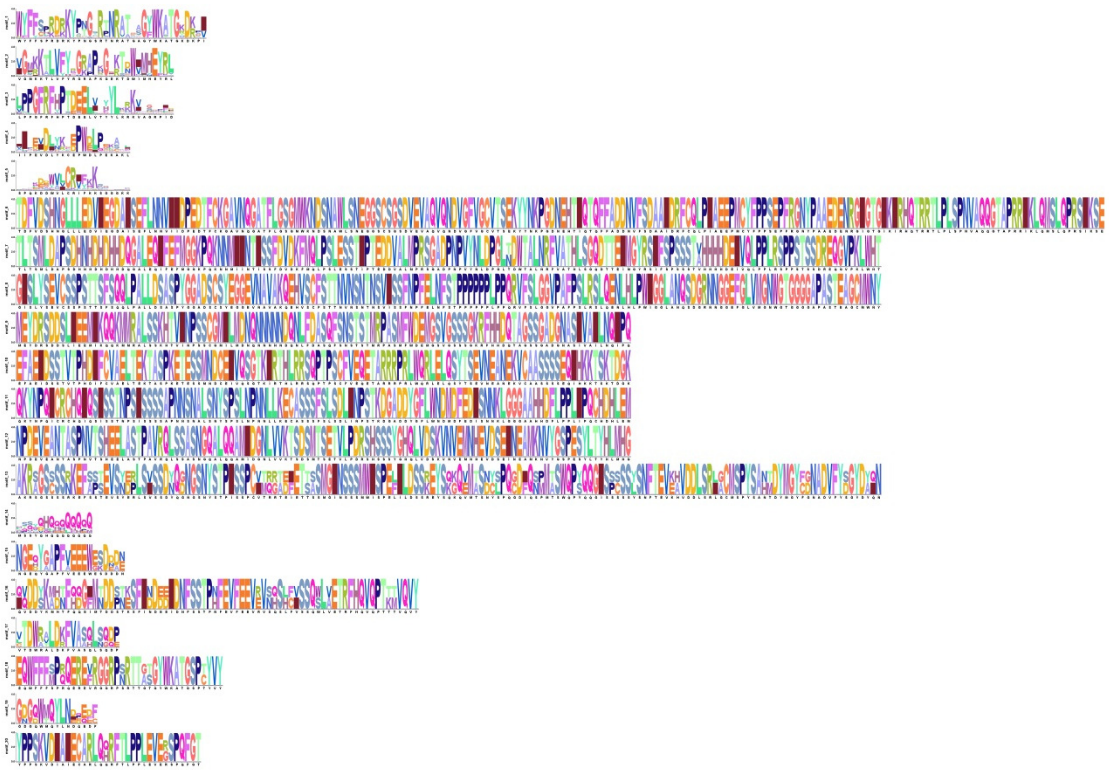

Figure S1. Multilevel consensus sequence and their logo of HuNAC proteins predicted by MEME web server.

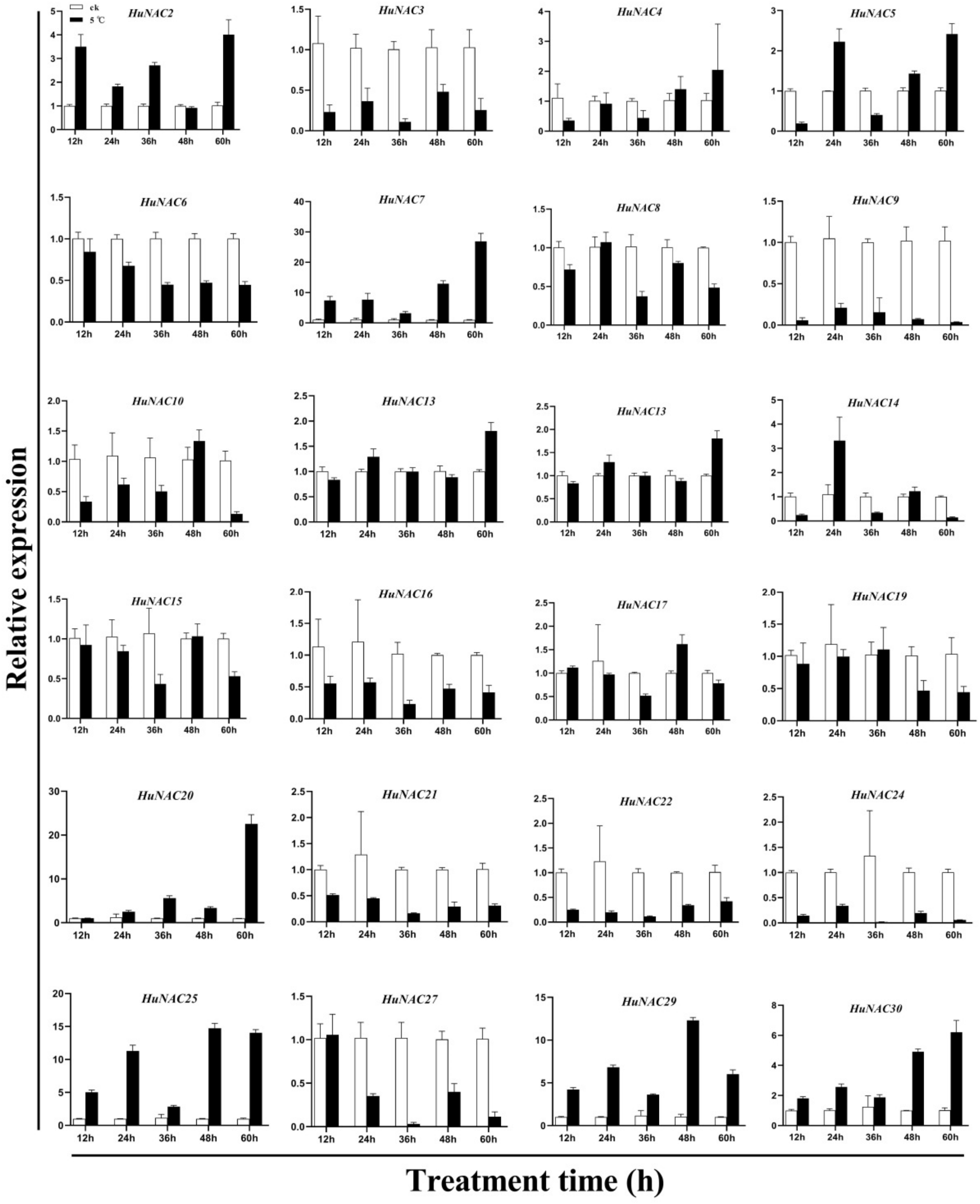
ck
5 ℃
HuNAC2
HuNAC3
HuNAC4
HuNAC5
HuNAC6
HuNAC7
HuNAC8
HuNAC9
HuNAC10
HuNAC13
HuNAC13
HuNAC14
HuNAC15
HuNAC16
HuNAC17
HuNAC19
HuNAC20
HuNAC21
HuNAC22
HuNAC24
HuNAC25
HuNAC27
HuNAC29
HuNAC30
12h
24h
36h
48h
60h
Relative expression
Treatment time (h)

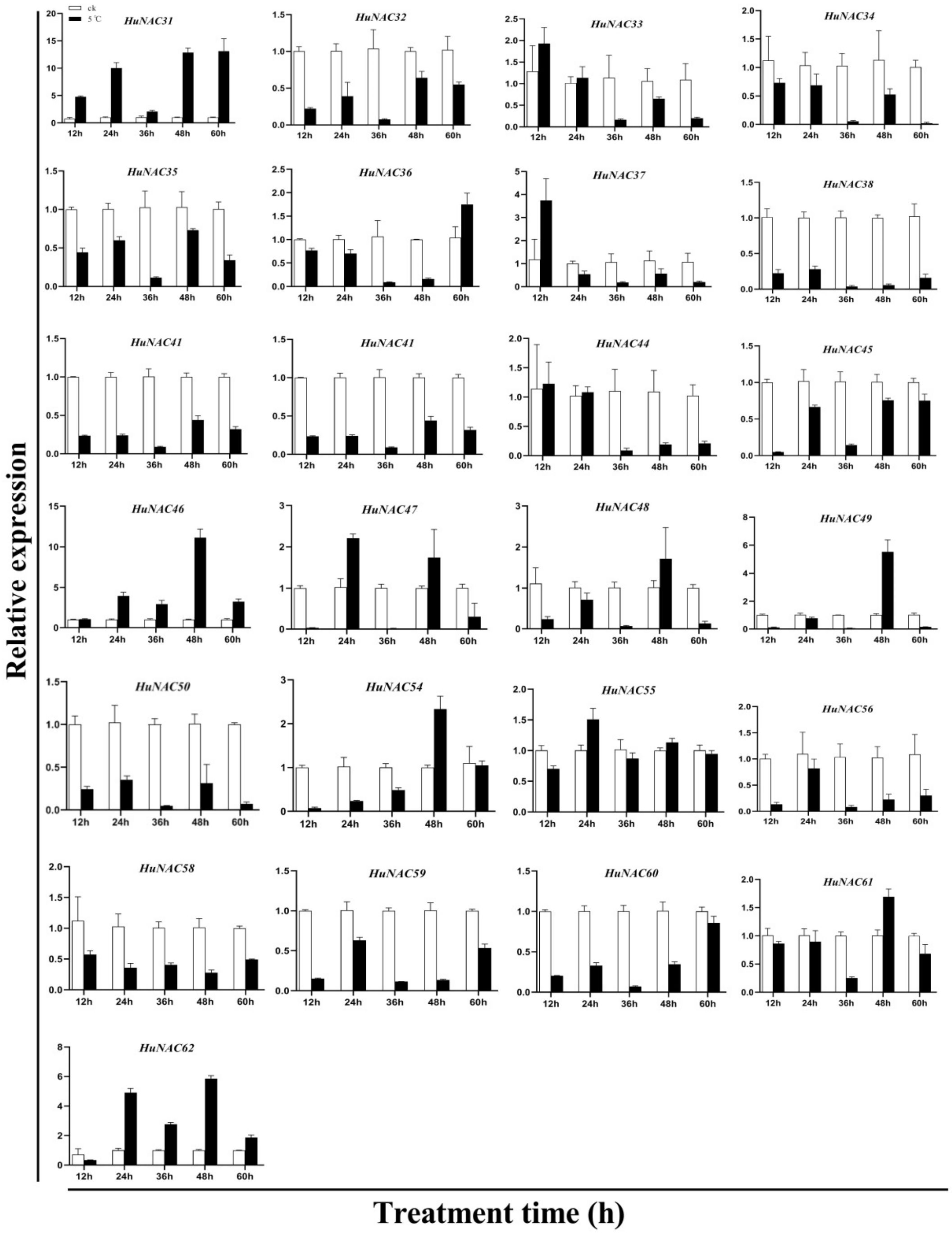

Figure S2. Expression patterns of 64 *HuNAC* genes under cold stress treatments. Three biological replicates were used and bars represent the relative expression of different genes under cold stress.

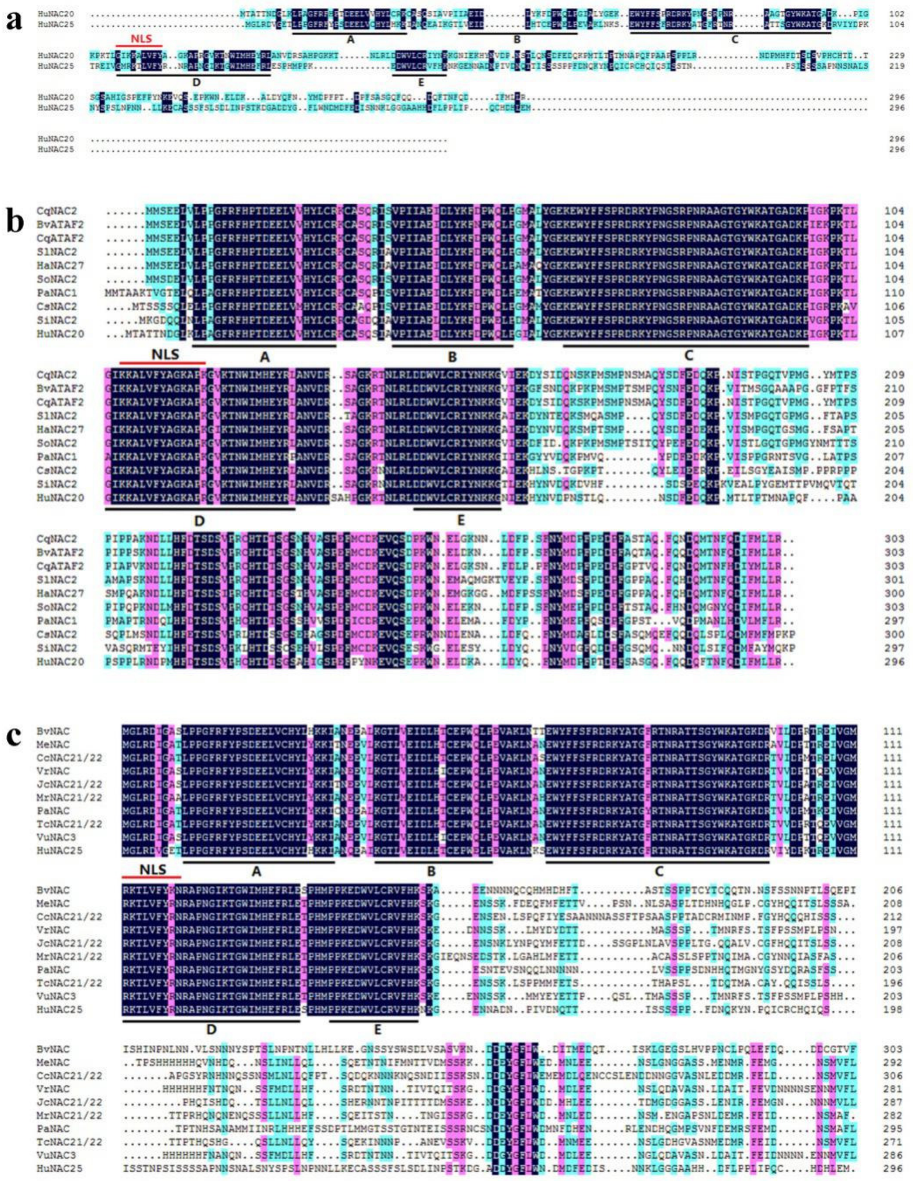

Figure S3. Multiple alignments of HuNAC20 and HuNAC25 proteins and other plant NAC proteins. The accession numbers are as follows: CqNAC2 (*Chenopodium quinoa*, XP_021772603.1), BvATAF2 (*Beta vulgaris*, QGZ00533.1), CqATAF2 (*Chenopodium quinoa*, XP_021734633.1), SlNAC2 (*Suaeda liaotungensis*, AGZ15313.1), HaNAC27 (*Haloxylon ammodendron*, AOC59210.1), SoNAC2 (*Spinacia oleracea*, XP_021863783.1), PaNAC1 (*Phytolacca acinosa*, ADQ00628.1), CsNAC2 (*Camellia sinensis*, XP_028092030.1), SiNAC2 (*Sesamum indicum*, XP_011099683.1), BvNAC (*Beta vulgaris*, XP_010679326.1), MeNAC (*Manihot esculenta*, XP_021630142.1), CcNAC21/22 (*Citrus clementine*, XP_006443820.1), VrNAC(*Vigna radiate var. radiate*, XP_014495173.1), JcNAC21/22 (*Jatropha curcas*, XP_012085297.1), MrNAC21/22 (*Morella rubra*, KAB1223758.1), PaNAC (*Parasponia andersonii*, PON62646.1), TcNAC21/22 (*Theobroma*

cacao, XP_007050267.1), VuNAC3 (*Vigna unguiculata*, XP_027920779.1)
