# Supplementary material for: HuNAC20 and HuNAC25, Two Novel NAC Genes from Pitaya, Confer Cold Tolerance in Transgenic Arabidopsis
Source: Int J Mol Sci. 2022 Feb 16;23(4):2189. doi: 10.3390/ijms23042189 (PMC8876859; doi:10.3390/ijms23042189)
Supplement: Supplementary file 1 [file ijms-23-02189-s001.zip › Table S1 All HuNAC genes identified from the pitay.pdf]

Table S1 All *HuNAC* genes identified from the pitaya genome

Chromosome	Rename	Gene ID	Protein length (aa)	Molecular weight (kDa)	Theoretical PI	Hydrophilicity index
Chromosome 1	*HuNAC1*	HU01G00060.1	353	40.16	5.11	-0.724
	HuNAC2	HU01G00420.1	306	35.05	8.71	-0.656
	HuNAC3	HU01G01336.1	279	30.95	8.65	-0.525
	HuNAC4	HU01G01551.1	214	24.80	9.25	-0.848
	HuNAC5	HU01G01910.1	355	39.45	9.06	-0.694
	HuNAC6	HU01G01912.1	191	22.00	9.56	-0.613
	HuNAC7	HU01G02030.1	315	36.03	6.37	-0.796
	HuNAC8	HU01G02105.1	323	36.44	6.33	-0.611
	HuNAC9	HU01G02216.1	244	28.07	6.67	-0.798
	HuNAC10	HU01G02747.1	155	18.12	9.73	-0.652
Chromosome 2	*HuNAC11*	HU02G00016.1	270	30.33	7.60	-0.784
	HuNAC12	HU02G00325.1	417	47.10	5.41	-0.611
	HuNAC13	HU02G00350.1	285	32.94	8.90	-0.847
	HuNAC14	HU02G01957.1	174	20.63	8.35	-0.656
	HuNAC15	HU02G03060.1	344	38.24	8.66	-0.670
Chromosome 3	*HuNAC16*	HU03G00363.1	300	34.23	5.42	-0.688
	HuNAC17	HU03G00832.1	571	63.93	4.82	-0.496
	HuNAC18	HU03G00963.1	243	27.71	5.46	-0.696
	HuNAC19	HU03G00965.1	612	69.35	5.44	-0.629
	HuNAC20	HU03G01381.1	296	33.74	7.69	-0.717
	HuNAC21	HU03G01415.1	305	34.57	4.78	-0.490
	HuNAC22	HU03G01419.1	339	38.30	4.90	-0.592
	HuNAC23	HU03G02275.1	338	38.46	6.85	-0.921
Chromosome 4	*HuNAC24*	HU04G00486.1	322	36.66	5.57	-0.684
	HuNAC25	HU04G01001.1	296	33.65	6.26	-0.692

	HuNAC26	HU04G01141.1	332	37.76	8.84	-0.717
	HuNAC27	HU04G01162.1	315	35.26	7.55	-0.659
	HuNAC28	HU04G01575.1	363	40.04	6.08	-0.553
	HuNAC29	HU04G01653.1	656	72.34	5.07	-0.620
	HuNAC30	HU04G01686.1	684	75.87	5.33	-0.557
	HuNAC31	HU04G01838.1	296	33.65	6.26	-0.692
Chromosome 5	*HuNAC32*	HU05G00302.1	329	37.15	5.71	-0.624
	HuNAC33	HU05G00497.1	368	42.23	6.59	-0.837
	HuNAC34	HU05G00603.1	344	37.76	8.11	-0.492
	HuNAC35	HU05G00958.1	494	55.25	6.73	-0.877
	HuNAC36	HU05G01179.1	312	35.49	5.86	-0.610
	HuNAC37	HU05G01191.1	391	43.96	8.54	-0.500
	HuNAC38	HU05G02103.1	366	41.73	5.27	-0.793
Chromosome 6	*HuNAC39*	HU06G00112.1	375	42.70	5.36	-0.783
	HuNAC40	HU06G01181.1	325	36.93	5.42	-0.624
	HuNAC41	HU06G01682.1	454	51.25	8.90	-0.627
Chromosome 7	*HuNAC42*	HU07G00181.1	353	40.07	6.39	-0.682
	HuNAC43	HU07G00186.1	350	39.81	6.39	-0.704
	HuNAC44	HU07G00263.1	319	36.96	5.38	-0.897
	HuNAC45	HU07G00854.1	302	34.53	6.76	-0.663
	HuNAC46	HU07G02058.1	282	31.46	7.08	-0.565
Chromosome 8	*HuNAC47*	HU08G00445.1	361	40.18	9.13	-0.618
	HuNAC48	HU08G00521.1	344	37.76	8.11	-0.492
	HuNAC49	HU08G00888.1	403	45.02	5.66	-0.691
	HuNAC50	HU08G01013.1	244	27.78	5.43	-0.725
	HuNAC51	HU08G01016.1	573	64.65	5.43	-0.725
	HuNAC52	HU08G01430.1	341	37.86	5.08	-0.540
	HuNAC53	HU08G02126.1	327	36.25	8.94	-0.588

	HuNAC54	HU08G02135.1	327	36.25	8.94	-0.588
Chromosome 9	*HuNAC55*	HU09G00056.1	154	17.95	9.19	-0.553
	HuNAC56	HU09G00230.1	393	44.19	6.81	-0.566
	HuNAC57	HU09G00238.1	289	32.24	5.89	-0.716
	HuNAC58	HU09G01575.1	328	36.17	8.89	-0.716
Chromosome 10	*HuNAC59*	HU10G00023.1	272	30.34	8.37	-0.656
	HuNAC60	HU10G00380.1	281	32.34	8.54	-0.741
	HuNAC61	HU10G01741.1	347	38.70	8.36	-0.627
Chromosome 11	*HuNAC62*	HU11G01180.1	299	34.19	6.67	-0.723
	HuNAC63	HU11G01542.1	331	38.33	5.95	-0.798
	HuNAC64	HU11G01605.1	377	42.84	5.67	-0.753
